# Differentiation therapy in acute myeloid leukaemia: molecular basis, clinical progress, and future perspectives

**DOI:** 10.3389/fmed.2026.1818483

**Published:** 2026-05-18

**Authors:** Abdulaziz Asiri

**Affiliations:** Department of Medical Laboratory Sciences, College of Applied Medical Sciences, University of Bisha, Bisha, Saudi Arabia

**Keywords:** acute myeloid leukemia (AML), acute promyelocytic leukemia (APL), all-trans retinoic acid (ATRA), arsenic trioxide (ATO), azacitidine, differentiation therapy, genomic profiling, IDH1/2 inhibitors

## Abstract

Clonal evolution, genetic heterogeneity, and impaired myeloid differentiation are characteristics of acute myeloid leukemia (AML), an aggressive hematologic malignancy. Many patients continue to have poor outcomes even after decades of relying on aggressive induction chemotherapy (7 + 3 regimen), particularly those who are elderly or have unfavorable molecular characteristics. Arsenic trioxide (ATO) and all-trans retinoic acid (ATRA) were effective treatments for acute promyelocytic leukemia (APL) which changed the therapeutic paradigm from cytotoxicity to maturation-based methods. More recently, differentiation therapy has been expanded to include other AML subtypes since IDH inhibitors were developed, (enasidenib, ivosidenib) and menin inhibitors (revumenib, ziftomenib). Meanwhile, epigenetic modulators like azacitidine emphasize the wider therapeutic impact of reversing transcriptional and metabolic blocks. Biomarker-driven stratification is now possible thanks to advancements in genomic profiling, which enhances the selection of patients who stand to gain the most from differentiation-based strategies. In addition to summarizing important therapeutic drugs and trial results, this review incorporates the most recent research on the molecular basis of differentiation arrest and looks at issues including resistance, relapse, and the absence of prognostic biomarkers. Differentiation therapy is a mechanistically sound and less harmful approach that supports targeted therapy and has the potential to redefine AML treatment by reestablishing normal hematopoietic development.

## Graphical abstract

**Figure:**
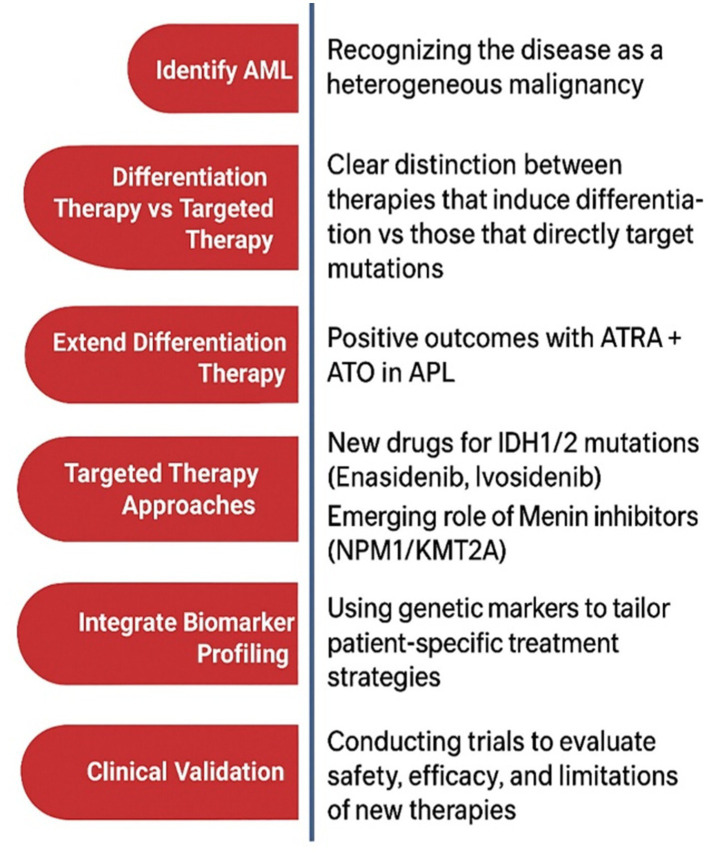

## 1 Introduction

Recent research has markedly improved our understanding of the genetic underpinnings of myeloid malignancies, offering insights into their pathophysiology and prospective therapies. Although often non-hereditary, these malignancies develop as clonal diseases resulting from acquired somatic mutations in hematopoietic progenitor cells. Initial insights were derived from the identification of recurrent chromosomal translocations associated with particular disease categories. Future advancements in genome sequencing have improved our ability to detect these mutations, resulting in increased diagnostic precision, prognosis evaluations, and focused therapeutic approaches (1).

AML is a complicated malignancy of the bone marrow and blood, most commonly diagnosed at later stages of life (2). Patients over the age of 65 face poor outcomes, AML has a 5-year survival rate below 20% (3). Our knowledge of AML can be enhanced by understanding chromosomal translocations like t(8;21), inv.(16), and t(15;17), as well as gene mutations like (*FLT3*, IDH1/2, TP53, CEBPA, and *NPM1*) may play key role in progression of disease (4, 5).

Additional fusion genes, such as RUNX1-RUNX1T1 in AML-M2 and PML-RARA in AML-M3 disrupt myeloid differentiation but fail to induce full leukemia in murine models without cooperating genetic events. This suggests that additional mutations or blocked cell differentiation are needed, highlighting the potential of therapies that target differentiation in specific leukaemia (6, 7).

The significance of genetic changes in leukemogenesis is likewise demonstrated by other myeloid cancers. For instance, the BCR-ABL1 fusion gene (t(9;22)) drives Chronic myeloid leukemia (CML), whereas cytopenias and recurrent mutations (such as 5q−, Ras, and C/EBPα) are present in myelodysplastic syndromes (MDS), some of which might lead to AML (8–10).

Differentiation therapy and targeted therapy must be conceptually differentiated, as they operate through distinct mechanisms. Differentiation therapy specifically addresses maturation impediments in leukemic blasts, reinstating terminal differentiation pathways that culminate in functional maturity and post-mitotic cell death. Conversely, targeted medicines suppress oncogenic drivers to affect proliferation, survival, or self-renewal signaling without directly activating differentiation programs. Differentiation treatment aims to restore the capacity for final differentiation in leukemic blasts by removing the maturation barrier. Notable instances include arsenic trioxide (ATO) and all-trans retinoic acid (ATRA) in acute promyelocytic leukemia, along with contemporary IDH1/2 inhibitors (ivosidenib, enasidenib) that promote differentiation in acute myeloid leukemia (AML) with an IDH mutation (1, 9, 10).

Conversely, targeted therapy directly obstructs pro-leukemic molecular circuits by inhibiting specific oncogenic signaling pathways or mutant driving proteins, exemplified by the BCL-2 inhibitor venetoclax and *FLT3* inhibitors (midostaurin, gilteritinib) (8–10). Despite some overlap due to the differentiation-inducing properties of certain targeted inhibitors, such as IDH and Menin inhibitors, their treatment philosophies remain distinct. It is imperative to elucidate this distinction to avert conceptual misinterpretation and to underscore the unique potential of differentiation-based therapies in AML (11–17).

The development of specific medications like IDH, BCL-2, and *FLT3* inhibitors (venetoclax) and antibody drug conjugates (gemtuzumab ozogamicin) has led to significant advancements in recent years, even though the 7 + 3 regimen (cytarabine + daunorubicin) still serves as the foundation of treatment (11–13, 16, 18, 19). The limit of increasing the intensity of treatment leads to toxicity. Additional research is necessary to determine the best way to use newly developed drugs such *FLT3*, BCL-2, and HDAC inhibitors in conjunction with chemotherapy. Although relapse and damage are still common, intensive chemotherapy combined with allogeneic stem cell transplantation heals about 60% of younger patients (20). Poor results are still associated with relapsed/refractory AML, with a mere few months’ median survival.

With the advancements in targeted medicines, individualized therapy is still hampered by the lack of trustworthy prognostic biomarkers (21, 22). Precision oncology trials, including NCI-MATCH and SHIVA, demonstrated limited benefits due to few actionable mutations and modest response rates (23–29).

Thus, this review’s goal is to give readers a comprehensive understanding of Acute Myeloid Leukemia (AML), with an emphasis on its molecular mechanisms, clinical treatment, drug resistance, and potential for new treatment strategies.

## 2 Acute myeloid leukaemia: mechanism and epidemiology

The myeloid cell line gives rise to acute myeloid leukemia (AML), a rapidly developing cancer marked by an excessive build-up of immature precursor cells (blasts) in the bone marrow and peripheral circulation. This overproduction impairs the formation of healthy blood components especially erythrocytes and platelets leading to a swift and severe marrow failure, which occurs more abruptly than in chronic forms of leukemia. Although remission can often be achieved through intensive chemotherapy, allogeneic hematopoietic stem cell transplantation is still the sole treatment available.

Additionally, individuals diagnosed with high or very high-risk myelodysplastic syndromes (MDS) often presenting with chronic anaemia, thrombocytopenia, and circulating blast cells are at a significantly greater risk of evolving into AML. Such cases require continuous monitoring and proactive intervention due to their unstable clinical course (30). The unregulated clonal development of immature myeloid cells, or blasts, inside the bone marrow compartment is a hallmark of Acute Myeloid Leukemia (AML). These malignant cells crowd out normal hematopoietic elements, thereby impairing healthy blood cell production. Considerable research has focused on understanding how these leukemic blasts interact with and influence the bone marrow microenvironment. Their rapid expansion is largely fuelled by diverse genetic mutations and chromosomal abnormalities. Clinically, this manifests as disrupted erythrocyte and platelet formation, due to inefficient erythropoiesis and mega-karyopoiesis, ultimately causing marrow failure (31).

**Figure 1:**
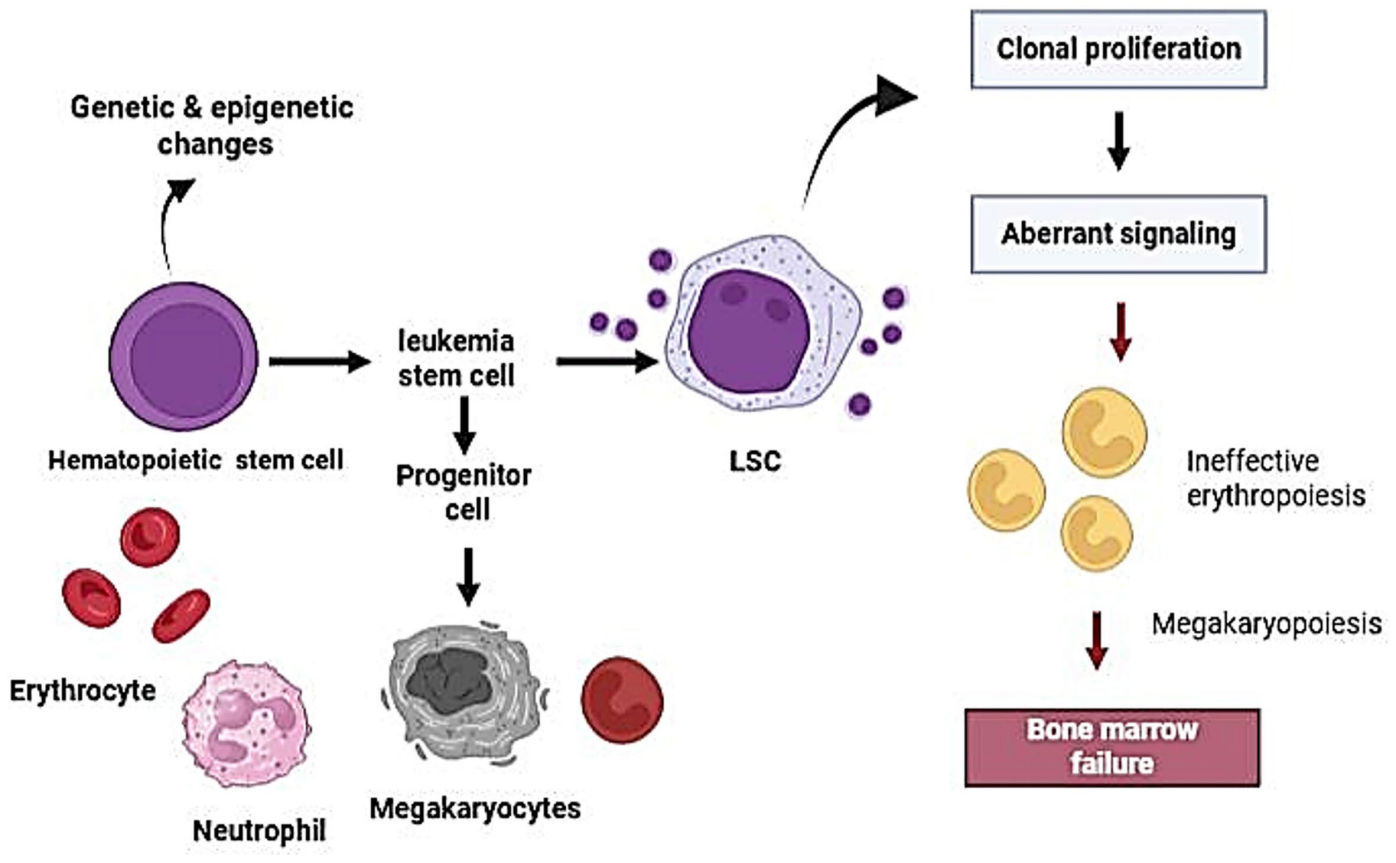
Leukemia stem cells (LSCs) are created when hematopoietic stem cells (HSCs) undergo genetic and epigenetic changes. These LSCs experience abnormal signaling and clonal proliferation, which leads to poor megakaryopoiesis, inefficient erythropoiesis, and eventually bone marrow failure. Crucially, this process also creates a differentiation block, which is a key pathogenic mechanism in acute myeloid leukemia (AML) and gives differentiation therapy its biological justification.

The aggregation of acquired genetic and epigenetic modifications in hematopoietic stem and progenitor cells (HSPCs) significantly contributes to the pathogenesis of Acute Myeloid Leukemia (AML). These alterations induce hematopoietic stem and progenitor cells (HSPCs) to shift from their standard phenotype and function to Leukemia stem cells (LSCs). Similar to conventional stem cells, these LSCs exhibit characteristics such as self-renewal, dormancy, and differentiation, which facilitate disease persistence and therapeutic resistance (Figure 1).

AML blasts occupy the pinnacle of a disordered hierarchy that reflects typical blood cell growth, a phenomenon repeatedly noted in AML patient samples (32). Recent investigations indicate that these blasts significantly interfere with regular hematopoiesis. Remarkably, AML patients frequently have normal or elevated quantities of hematopoietic stem cells (HSCs), while hematopoiesis is impaired. This arises in part from AML blasts expressing receptors such as the myeloproliferative leukemia protein and the thrombopoietin scavenging receptor, which induce neutropenia and thrombocytopenia. Exosomes formed from AML, which include microRNAs, further diminish HSC functionality, resulting in a widespread inhibition of healthy blood cell production (33). Despite advancements in therapy, overall survival remains suboptimal, especially among older patients (34–36).

### 2.1 Molecular mechanisms of differentiation arrest in AML

#### 2.1.1 Transcriptional dysregulation

Transcriptional dysregulation is essential for leukemic blast differentiation arrest in AML. Important transcription factors including RUNX1, CEBPA, and KAT6A are frequently altered or expressed abnormally, which disrupts gene expression programs that are necessary for healthy hematopoiesis (37–40). Leukemic stem cells may be prevented from differentiating and continuing to self-renew if, for example, alterations in RUNX1 affect its capacity to bind DNA and control target gene (1). Likewise, mutations in CEBPA are linked to a failure to start granulocytic differentiation, which leads to the buildup of immature myeloid cells. These transcriptional changes prolong the malignant cells’ undifferentiated status, which promotes leukemogenesis in addition to impeding normal cell maturation (39, 40).

#### 2.1.2 Epigenetic roots of AML

Multiple different sub-clonal populations are present at diagnosis in Acute myeloid leukemia (AML), a genetically diverse malignancy. These clones frequently retain mutations in epigenetic regulators such *TET2, DNMT3A*, and *ASXL1*, which continue through remission and relapse, indicating their early involvement in leukemogenesis and contribution to the growth of pre-leukemic hematopoietic stem and progenitor cells (HSPCs). These mutations impair normal epigenetic control, resulting in defective cellular differentiation, a key characteristic of both AML and Myelodysplastic Syndromes (MDS) (41–45).

#### 2.1.3 Metabolic and stem cell regulation

In AML, metabolic reprogramming is another important factor that leads to differentiation arrest. The metabolic pathways of leukemic stem cells (LSCs) are modified to promote their survival and growth. *IDH1* and *IDH2* mutations, for instance, produce the oncometabolite 2-hydroxyglutarate (2-HG), which prevents histone and DNA demethylases from working, causing epigenetic modifications that prevent differentiation (15, 46). Certain inhibitors, such as Ivosidenib for *IDH1* mutations and Enasidenib for *IDH2* mutations, have demonstrated promise in restoring normal differentiation in AML cells by targeting these metabolic pathways (15). These metabolic changes also affect the bone marrow niche, which promotes LSC survival and increases the likelihood of illness recurrence and persistence (46).

### 2.2 Clinical symptoms and progressions of AML

Recurrent infections, anaemia-related exhaustion, easy bruising, prolonged bleeding, bone pain, headaches, and widespread discomfort because of compromised erythropoiesis and bone marrow failure are common symptoms of acute myeloid leukemia (AML). Weakness, chest pain, or dyspnoea can all be symptoms of severe anaemia. It usually takes days or weeks for symptoms to appear suddenly. Patients frequently exhibit pallor, bruises, and possibly hepatosplenomegaly upon examination; lymphadenopathy is rare.

Some patients may develop myeloid sarcoma, which manifests as skin sores or isolated extramedullary tumors. Leukostasis, disseminated intravascular coagulation (DIC), and tumor lysis syndrome (TLS) are serious outcomes of AML which can show up as neurological impairments, hemorrhage, purpura, renal dysfunction, or respiratory compromise. Spontaneous TLS is extremely rare and needs to be treated right away before treatment (47, 48, 151). If treatment is not received, leukemic blasts build up and AML advances quickly, leading to severe marrow failure in a matter of weeks. Flow cytometry and immunophenotyping are usually used to demonstrate that 20% or more of the bone marrow’s nucleated cells or blood are blasts, which is a definite diagnosis (49).

### 2.3 Disease burden and challenges in treatment

Between 1990 and 2021, the number of reported AML cases rose significantly from 79,372 to 144,645 likely due to factors such as global population growth, aging demographics, and advancements in diagnostic methods (152). Disability-adjusted life years (DALYs) and the age-standardized death rate (ASDR) have declined despite an increase in AML-related deaths worldwide. This discrepancy suggests that while the number of diagnoses is rising, the disease’s consequences on people may have been lessened by improved treatments and healthcare administration. The decline in age-standardized DALYs adds credence to the population’s overall decline in the burden of specific diseases (50).

#### 2.3.1 Burden in high SDI vs. low SDI countries

There are notable differences in the prevalence of AML among various sociodemographic index (SDI) regions, according to global epidemiological data. Improved diagnostic capabilities, increased knowledge of the disease, and extensive cancer registries are largely responsible for the highest rises in incidence and age-standardized incidence rates (ASIR) seen in high-SDI countries. Increased prevalence in these areas is also a result of lifestyle-related variables, obesity, and environmental pollution (51). High-SDI regions continue to report the highest AML-related mortality and disability-adjusted life years (DALYs) despite sophisticated healthcare systems, which is indicative of the disease’s aggressive nature. On the other hand, tobacco use, environmental exposures like benzene, and genetic predispositions propel rising incidence rates in middle- and low-SDI regions; nevertheless, their disease outcomes are further worsened by a lack of access to innovative medications and a lack of suitable healthcare infrastructure (52, 53).

#### 2.3.2 Aging and high mortality in elderly

Age has a significant impact on the occurrence of AML, and mortality rates for those diagnosed after age 65 surpass 90% (52). Leukemogenesis is predisposed to and treatment tolerance is limited by aging-related factors such as immune-senescence, clonal haematopoiesis, cumulative genomic damage, and decreased bone marrow regeneration potential (54). Comorbidities and frailty also limit access to rigorous chemotherapy and stem cell transplantation, which means that many older patients must use hypomethylating drugs or low-intensity regimens that only produce moderate results (55). Because they continue to be the most vulnerable demographic, older individuals account for the largest percentage of AML-related mortality globally.

#### 2.3.3 Major risk factors of AML

**Figure 2:**
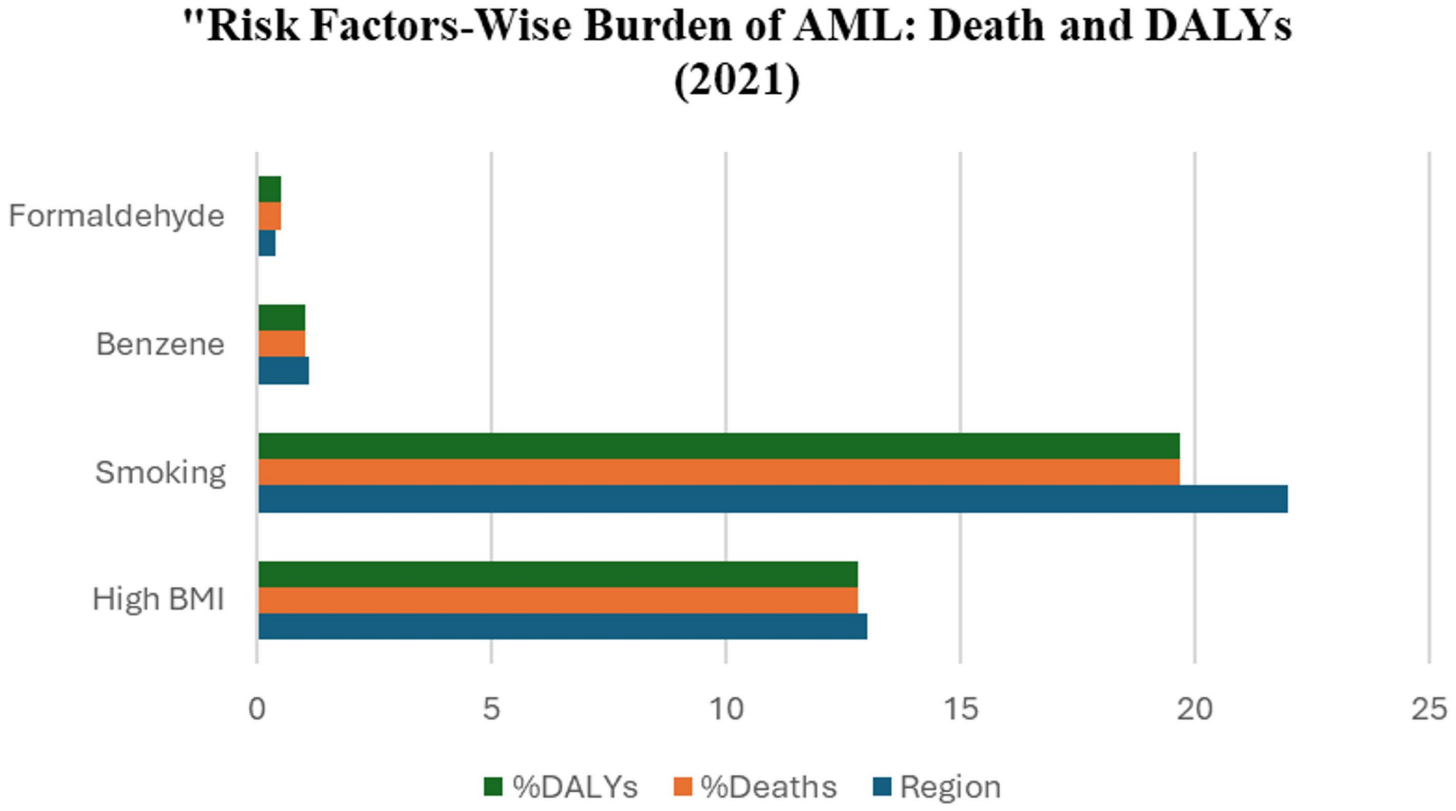
Contribution of major risk factors to AML-related deaths and DALYs in 2021. Smoking and high BMI are the leading contributors, particularly in high-income regions, while benzene and formaldehyde exposure play smaller but notable roles, especially in developing countries.

The pathophysiology of acute myeloid leukemia (AML) has been linked to a number of modifiable and environmental risk factors. Increased body mass index (BMI), occupational exposure to hematotoxic chemicals, and tobacco use are all consistently correlated (Figure 2). Occupational exposure to hazardous chemicals, particularly benzene and formaldehyde, is a well-established risk factor for haematological malignancies, including acute myeloid leukaemia (AML). Benzene, extensively utilised in industrial applications, is recognised for causing bone marrow suppression and chromosomal abnormalities in haematopoietic stem cells. Formaldehyde, an industrial chemical, has been associated with DNA damage and clonal haematopoiesis. Workers in sectors such as petrochemicals, printing, rubber production, and agriculture face heightened risk, particularly in environments with insufficient safety rules or extended exposure. Further, an increased BMI is associated with persistent low-grade inflammation, modified cytokine profiles, and insulin resistance, all of which may lead to genomic instability and facilitate leukemogenesis. Epidemiological studies indicate that individuals with obesity may possess a slightly elevated chance of acquiring acute myeloid leukaemia, especially in the absence of additional predisposing factors. Tobacco smoking on the other hand is a well-established and modifiable risk factor for AML among lifestyle-related causes. Cigarette smoke comprises numerous carcinogenic substances, such as benzene, polycyclic aromatic hydrocarbons (PAHs), nitrosamines, and formaldehyde, which can cause DNA damage, epigenetic alterations, and mutations in haematopoietic cells. Multiple meta-analyses and pooled cohort studies have repeatedly demonstrated that both present and former smokers possess a markedly elevated risk of acute myeloid leukaemia (AML) in comparison to never smokers (56).

### 2.4 Treatment challenges and resistance

The traditional induction therapy known as the “7 + 3 regimen” (cytarabine plus an anthracycline) has been the mainstay of treatment for Acute Myeloid Leukemia (AML) since the 1970s. Significant problems still exist, such as primary chemoresistance, high relapse rates, and poor tolerance in elderly or unfit persons, even though this regimen produces remission in many younger and fit patients (4, 5, 57, 58). The approval of targeted treatments during the past 10 years has changed therapeutic paradigms. *IDH1/2* inhibitors (ivosidenib, enasidenib), *BCL-2* inhibitor venetoclax (in conjunction with hypomethylating agents), *FLT3* inhibitors (midostaurin, gilteritinib), and the antibody–drug conjugate gemtuzumab -ozogamicin have increased treatment options, especially for patients with genetically predisposed to adverse outcomes or those who cannot receive intensive chemotherapy (11, 14, 15).

These medicines, which are now used in frontline or relapsed/refractory treatment settings based on patient fitness and genetic profile, have drastically changed clinical practice. Despite these developments, clonal evolution and treatment resistance continue to be significant challenges. According to clinical data, up to 30–40% of patients’ relapse or are resistant to venetoclax-based regimens. Adaptive signalling, mitochondrial reprogramming, and the selection of resistant subclones are some of the resistance mechanisms (59). Personalized treatment selection is currently limited by the absence of predictive biomarkers, despite the fact that targeted medicines have improved results, according to patient’s prognosis (60). Therefore, in order to attain more profound and long-lasting remissions, new differentiation-based approaches and precision-guided treatments are desperately needed (61).

## 3 Genomic profiling in AML and its implication in targeted therapy

Due to their ability to facilitate diagnosis, risk assessment, prognosis, treatment selection, and disease progression monitoring biomarkers are essential in treatment of Acute Myeloid Leukemia (AML) (37, 38). AML subtypes are categorized and targeted therapies are guided by important chromosomal translocations, gene mutations, and cell surface indicators that have been discovered by advances in molecular genetics (39, 40). The European LeukemiaNet (ELN) and the National Comprehensive Cancer Network (NCCN) have created risk classification frameworks that use cytogenetic and molecular abnormalities to predict outcomes, direct therapy choices, and enhance patient care (37, 38, 62).

Additionally, a large number of biomarkers function as indications of measurable residual disease (MRD), which enables physicians to track therapy response and predict recurrence (41).

The choice and efficacy of differentiation therapies are significantly influenced by particular genomic changes; for instance, responsiveness to all-trans retinoic acid (ATRA) is predicted by PML-RARA translocations, demonstrating how genomic profiling can directly inform differentiation-based treatment approaches (17). In order to set the stage for a thorough examination of particular chromosomal translocations, immunophenotypic markers, and recurrent gene mutations, this section highlights crucial cytogenetic, immunophenotypic, and molecular biomarkers in AML. It also highlights their potential role in directing differentiation therapy.

### 3.1 Chromosomal translocations and fusion genes

#### 3.1.1 PML-RARA (t(15;17))

A characteristic of Acute Promyelocytic Leukemia (APL), the PML-RARA fusion gene is found in approximately 98% of patients due to the t(15;17) chromosomal translocation (63). This fusion protein drives APL by disrupting normal gene transcription, particularly processes critical for cell differentiation (64). All-trans Retinoic Acid (ATRA) and Arsenic Trioxide (ATO) are two common APL medications that patients with the PML-RARA fusion usually respond well. The combination of ATRA and ATO produces exceptional outcomes in low-risk APL patients, with 100% full remission rates and roughly 97% event-free survival rates at 2 years (65–67). Diagnostic tools such as Fluorescence *in situ* Hybridization (FISH) and real-time PCR are effective for detecting PML-RARA fusions and monitoring minimal residual disease (MRD) during follow-up (144). APL is associated with over ten distinct RARA fusion variants, including *ZBTB16-RARA*, *STAT5B-RARA*, and *NPM1*-RARA, many of which exhibit poor responsiveness to ATRA and ATO, marking them as key indicators of treatment resistance. Identifying these variants is essential for accurate diagnosis, prognosis, and tailoring therapeutic strategies (68, 69).

#### 3.1.2 AML1-ETO (t(8;21))

The chromosomal rearrangement t(8;21) (q22;q22) results in the AML1-ETO (*RUNX1-RUNX1T1*) fusion gene, a prominent cytogenetic abnormality in AML, notably linked to core binding factor (CBF)-AML. This fusion protein acts as a transcriptional suppressor, inhibiting genes typically controlled by AML1, it causes leukemogenesis, accumulates immature myeloid cells, and interferes with normal hematopoietic differentiation. This translocation, which is most prevalent in the M2 subtype and uncommon in the M1 and M4 subtypes, is present in around 5–10% of all AML cases and 7–12% of adult cases reflecting subtype specificity.

Individuals with t(8;21)-positive AML generally experience a favorable prognosis, with elevated remission rates and prolonged overall survival (OS), especially in the absence of additional high-risk mutations, and they typically respond well to standard chemotherapy regimens (154). Sensitive methods such as reverse transcription quantitative polymerase chain reaction (RT-qPCR) and fluorescence in situ hybridization (FISH) are used to identify the AML1-ETO fusion and monitor minimal residual disease (MRD) during and after treatment (70, 71). Additionally, about 20–25% of newly diagnosed AML cases, including those with t(8;21), include c-Kit gene mutations, which may worsen outcomes and reclassify patients into the intermediate-risk group according to National Comprehensive Cancer Network (NCCN) criteria (154).

### 3.2 Immunophenotypic markers

#### 3.2.1 CD34 expression

The glycoprotein CD34 is found on the surface of progenitor and hematopoietic stem cells has been extensively studied in AML. Research indicates that 66.7% of AML patients exhibit CD34 expression, which is significantly associated with unfavorable cytogenetic features, such as deletions in chromosomes 5 or 7 and the t(8;21) translocation, while APL cases harboring t(15;17) are typically CD34-negative. CD34 positivity is more prevalent in less differentiated FAB subtypes, such as M0, M1, and M2. Detection is performed via flow cytometry, with positivity defined as ≥10% of blast cells showing staining. Clinically, CD34-positive patients demonstrate shorter overall and disease-free survival and lower rates of full remission.

Multivariate analysis identifies CD34 expression as an independent negative prognostic indicator, regardless of other factors, highlighting its dual role as a diagnostic and prognostic biomarker in AML (72).

### 3.3 Recurrent gene mutations in AML

#### 3.3.1 *NPM1* mutation

The *NPM1* gene, positioned on chromosome 5, produces a chaperone protein vital for DNA repair, ribosome synthesis, and p53 regulation. *NPM1* mutations result in its aberrant cytoplasmic localization, contributing to AML development, especially in M4 and M5 subtypes. About 30% of AML cases had these mutations, which are associated with higher WBC counts, decreased CD34 expression, and 58–60% complete remission (CR) rates. *NPM1* mutations are found using real-time quantitative polymerase chain reaction (RT-qPCR), which facilitates minimal residual disease (MRD) monitoring both during and after treatment.

According to guidelines from the National Comprehensive Cancer Network (NCCN) and European LeukemiaNet (ELN), allogeneic hematopoietic stem cell transplantation (allo-HSCT) is usually not recommended after initial remission, and AML with *NPM1* mutations, in the absence of *FLT3*-ITD mutations or with a low *FLT3*-ITD allelic burden, is associated with a favorable prognosis. Treatment with all-trans retinoic acid (ATRA) and arsenic trioxide (ATO) may increase mutant *NPM1* degradation, but further study is required to validate this strategy. Elevated *NPM1* levels in peripheral blood may indicate an approaching relapse (73).

#### 3.3.2 *FLT3* mutation (ITD and TKD)

The *FLT3* gene, which codes for a type III receptor tyrosine kinase (RTK), is located on the long arm of chromosome 13 that regulates hematopoietic cell proliferation, differentiation, and apoptosis via its extracellular ligand-binding domain. The most common RTK mutations in AML are *FLT3* mutations, which occur in around 30% of patients. Tyrosine kinase domain (*FLT3*-TKD) mutations account for about 7%, while internal tandem duplications (*FLT3*-ITD) account for about 25%. *FLT3*-ITD mutations are frequently connected to poor prognosis and normal karyotype AML. Modified *FLT3* continually activates the *PI3K, RAS/MAPK,* and *STAT5* pathways at the molecular level, improving the growth and survival of leukemic cells. Clinically, *FLT3*-mutated AML is characterized by leukocytosis, increased myeloid progenitor cells, and unfavorable outcomes (37, 38, 41, 155).

*FLT3*-ITD mutations are more severe than *FLT3*-TKD point mutations, correlating with reduced overall survival (OS) and event-free survival (EFS). The ratio of mutant to wild-type *FLT3* alleles significantly influences prognosis, with higher ratios predicting worse outcomes, and DNA fragment analysis is used to measure this ratio for risk stratification. *FLT3*-ITD mutations rarely coexist with complex karyotypes or core binding factor (CBF)-AML but may appear alongside PML-RARA rearrangements (74). Therapeutically, *FLT3* inhibitors like sorafenib, midostaurin, and sunitinib show clinical benefit, particularly in combination with chemotherapy. While resistance often limits monotherapy, combined treatment strategies have become standard for managing *FLT3*-mutated AML (75).

#### 3.3.3 *TP53* gene targets

The *TP53* gene, situated on chromosome 17p, translates a transcription factor crucial for DNA repair and cell cycle regulation. Missense mutations, the most common type, disrupt its tumor-suppressive function, leading to uncontrolled cell proliferation. Although less prevalent in hematologic malignancies than in solid tumors, about 10% of newly diagnosed AML cases, 20–37% of therapy-related AML cases, and up to 70% of AML cases with complicated karyotypes—especially in older patients or those with chromosomal 5, 7, or 17p abnormalities—have *TP53* mutations (76). These mutations are strongly linked to an unfavorable prognosis, with a complete remission (CR) rate of only 28.6%, even following hematopoietic stem cell transplantation (HSCT), there is a greater chance of relapse and a median overall survival of 6–8 months.

Conventional chemotherapy often shows limited effectiveness, but some trials suggest improved outcomes with a 10-day decitabine regimen for TP53-mutated AML. Furthermore, the tiny chemical APR-246 (eprenetapopt) recovers the structure and function of wild-type *p53* has shown promising results in early-phase clinical trials. When combined with azacitidine, this therapy achieved CR rates of about 82%, highlighting its potential as a targeted treatment for *TP53*-mutated AML and myelodysplastic syndromes (MDS). European Medicines Agency (EMA) has designated this combination as an orphan medicine and the U. S. Food and Drug Administration (FDA) for AML and MDS. Given its clinical significance, the European LeukemiaNet (ELN) guidelines advocate for early TP53 mutation testing to enhance risk stratification and guide treatment planning (77, 78).

#### 3.3.4 *IDH1/2* mutations in AML

*IDH1* and *IDH2* genes, positioned on chromosomes 2 and 15, respectively, encode tumor suppressor enzymes critical for cellular metabolism and energy production. *IDH1* operates in the cytoplasm, whereas *IDH2* functions within mitochondria. Mutations in these genes cause the buildup of 2-hydroxyglutarate, resulting in hypermethylation of DNA and histones, which hinders hematopoietic cell differentiation and drives leukemogenesis (79).

These mutations are constant across age groups, occurring in 15–20% of AML patients and 25–30% of AML cases with normal karyotypes. They often coexist with *NPM1* mutations but are infrequently found with *FLT3*-ITD, *TET2,* or *WT1* mutations, likely due to overlapping effects on DNA methylation (79). AML with *IDH1/2* mutations typically exhibits low white blood cell counts and elevated platelet levels, with prognostic implications. Some studies link *IDH1/2* mutations to reduced relapse-free survival (RFS) and overall survival (OS) (80–82). While others suggest a protective effect in *NPM1*-mutated AML (17). Beyond diagnostics, *IDH1/2* mutations serve as reliable markers for minimal residual disease (MRD) and can predict relapse when detected through sequencing (42). Targeted therapies have been developed to address these mutant enzymes Ivosidenib (AG-120), an *IDH1* inhibitor, reduces 2-hydroxyglutarate levels and promotes cell differentiation, receiving FDA approval in 2018 for relapsed or refractory *IDH1*-mutant AML (153). Similarly, enasidenib (AG-221), an IDH2 inhibitor, was approved in 2017 for relapsed or refractory *IDH2*-mutant AML (83). Both drugs are under active investigation, particularly in combination with cytarabine-based chemotherapy regimens.

#### 3.3.5 Molecular interplay between co-occurring mutations

*NPM1* mutations typically co-occur with *FLT3-*ITD in approximately 40–50% of *NPM1*-mutated AML patients and with *IDH1/2* in about 20–30%, resulting in unique molecular subtypes that influence AML biology through synergistic signaling and epigenetic alterations (84, 85). *NPM1c* (mutant NPM1) mislocalizes to the cytoplasm, impairing nucleolar function and ARF suppression, whereas *FLT3*-ITD induces ligand-independent kinase activation through *STAT5/PI3K/MAPK*, facilitating proliferation and inhibiting differentiation. Their synergy expedites AML onset (short latency in murine models), augments clonal dominance by allele amplification, and elucidates frequent co-occurrence; yet, a high *FLT3*-ITD allelic ratio deteriorates prognosis (86, 87). On the other hand, the interplay between *NPM1*and *IDH1/2* leads to another molecular mechanism, *IDH1/2* mutations generate the oncometabolite 2-HG, which inhibits *TET2/DNMT3A*, leading to DNA hypermethylation and obstructing differentiation; *NPM1c* further contributes by modifying HOX gene expression and ribosome biogenesis. Co-mutated acute myeloid leukemia (AML) exhibits mature/monocytic characteristics, increased *NPM1/IDH2R140* enrichment, and superior outcomes compared to *NPM1/FLT3-ITD*, with *IDH* co-mutation mitigating the detrimental effects of *FLT3*-ITD in triple-mutated patients (88). These pairings transition AML towards stem-like (NPM1/FLT3-ITD: immature blasts, low differentiation) or differentiated phenotypes (NPM1/IDH: enhanced response potential), affecting immune evasion and relapse by epigenetic reprogramming (89). The treatment may also impacted due to these combined mutation and it has been noted that *NPM1/FLT3*-ITD exhibits resistance to chemotherapy (increased relapse rates) but responds favorably to *FLT3* inhibitors (e.g., quizartinib) and menin inhibitors; *IDH* inhibitors (ivosidenib/enasidenib) demonstrate superior efficacy in *NPM1/IDH* subsets, promoting differentiation, although they are less effective with *NPM1c* compared to *FLT3*-ITD combinations—necessitating subtype-specific clinical trials (88).

## 4 Differentiation therapy in AML

Acute Myeloid Leukemia (AML) is a diverse blood malignancy marked by impaired differentiation of myeloid progenitors, resulting in unregulated clonal proliferation. Numerous genetic and epigenetic alterations disrupt normal myeloid maturation, resulting in stalled development and sustained self-renewal of leukemic cells. Despite advances in understanding AML’s molecular mechanisms, its primary treatment continues to depend on chemotherapy regimens developed in the 1970s (90). Patients with AML aged over 60 experience poor prognoses, as intensive chemotherapy offers minimal survival advantages mainly for individuals with good ELN genetics, whilst those with adverse genetics achieve cure rates of merely 10–15%, hence requiring experimental treatments (91). Since the 1970s, differentiation-based therapy has gained recognition as an innovative and effective method for managing Acute Myeloid Leukemia (AML) and various other malignancies. However, its clinical adoption did not occur until the late 1980s and even then, it was restricted to a specific subtype of AML, identified as acute promyelocytic leukemia (APL). Present-day treatment protocols for APL commonly utilize a dual therapy of all-trans-retinoic acid (ATRA) and arsenic trioxide, which function by disrupting the PML/RARα fusion protein that impedes cellular differentiation. This treatment has served as a benchmark example of how differentiation therapy can be successfully implemented in oncology (92). However, differentiation therapy successfully promotes maturity in bulk leukemic blasts but frequently does not eliminate leukemia stem cells (LSCs), permitting LSC persistence and facilitating relapse in acute myeloid leukemia (AML) (93). In acute promyelocytic leukemia (APL), all-trans retinoic acid (ATRA) promotes blast differentiation and diminishes leukemic stem cell (LSC) activity by decelerating replication; however, extended treatment is necessary for potential eradication, as cessation may lead to de-differentiation and relapse. In non-APL AML, treatments such as IDH inhibitors facilitate blast differentiation but encounter resistance driven by leukemic stem cells through stemness programs, underscoring inadequate targeting of leukemic stem cells (94, 95). Literature substantiates the superior bulk blast response of differentiation therapy compared to LSCs, although it is deficient in extensive head-to-head studies regarding LSC persistence measures (e.g., serial xenotransplants following therapy). Mouse studies demonstrate that therapy-induced mature AML lineages, such as eosinophils, endure during remission and contribute to relapse, highlighting the necessity for LSC-targeted combination therapies (96).

### 4.1 Concept of converting malignant blasts into mature functional cells

A hallmark of leukemia cells is their inability to progress beyond early developmental stages, preventing differentiation into mature, functional blood cells. In the 1970s and 1980s, researchers introduced a novel approach: promoting cancer cell differentiation and triggering their programmed cell death, offering an alternative to toxic chemotherapy. Early studies suggested that compounds such as dimethyl sulfoxide (DMSO) could induce erythropoiesis (97). Researchers commenced the identification of several substances that can direct the differentiation of myeloid leukaemia cells (98). A significant early discovery was the differentiation-inducing capability of All Trans Retinoic acid (ATRA). Various compounds including phorbol esters, teleocidins, planar polar drugs, cytokines, retinoids, and vitamin D analogs were found to significantly induce differentiation in leukemia cell lines like HL-60, KG-1, ML-3, and K562 in laboratory settings. These discoveries strengthened the idea of using differentiation-based strategies to treat cancers (149).

### 4.2 Key differentiating agents and their mechanistic actions in AML

**Figure 3:**
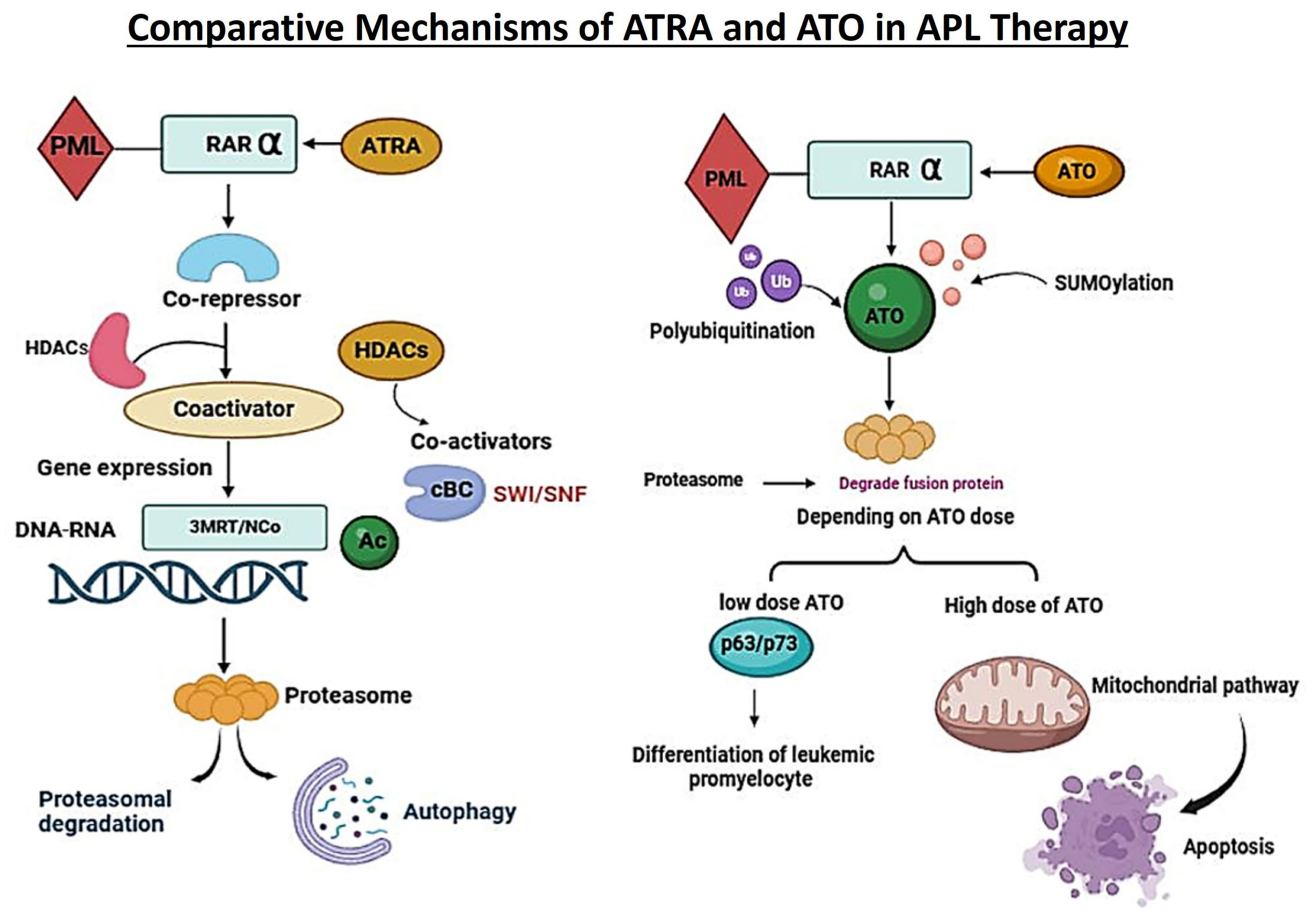
This illustration contrasts the molecular actions of all-*trans* retinoic acid (ATRA) and arsenic trioxide (ATO) in targeting the PML-RARα fusion protein in acute promyelocytic leukemia. ATRA promotes differentiation via gene transcription modulation, while ATO induces proteasomal degradation through polyubiquitination and SUMOylation. Depending on dose, ATO leads to leukemic differentiation or apoptosis via the mitochondrial pathway.

Acute myeloid leukemia (AML) treatment now includes differentiation-inducing medications in addition to traditional cytotoxic chemotherapy especially for specific molecular subtypes. In contrast to traditional chemotherapeutics that focus on eliminating proliferating leukemic blasts, differentiation therapy aims to promote terminal maturity of leukemic progenitors, thereby reinstating normal haematopoiesis and diminishing leukemic burden with potentially reduced toxicity (Figure 3; Table 1).

**Table 1:** Key differentiation -inducing agents used in the treatment of acute myeloid leukaemia (AML) and acute promyelocytic leukaemia (APL) highlighting their molecular targets, relevant biomarkers and clinical trial data including phase and identifiers.

Drug name	Target disease	Biomarkers	Clinical trial number	Phase
ATRA	Acute Promyelocytic Leukemia (APL)	PML-RARα fusion protein	ISRCTN55675535	Phase 3
ATRA	Acute Promyelocytic Leukemia (APL)	PML-RARα fusion protein	NCT00749728	Phase 3
Arsenic trioxide	Acute Promyelocytic Leukemia (APL)	PML-RARα, SUMO, ubiquitin, p53, p73	Included with ATRA trials	Phase 3
Enasidenib	IDH2-mutated AML (Relapsed/Refractory)	IDH2 mutation, 2-HG levels, IDH2 VAF	NCT01915498	Phase 1/2
Enasidenib	Post-Allo-SCT IDH2-mutated AML	IDH2 mutation, 2-HG levels, IDH2 VAF	NCT04522895	Phase 2
Ivosidenib (AG-120)	IDH1-mutated AML	IDH1 R132H/C mutations, 2-HG levels	NCT03173248	Phase 3
Ivosidenib (AG-120)	IDH1-mutated AML	IDH1 R132H/C mutations, 2-HG levels	NCT02074839	Phase 1
Azacitidine	AML (esp. with IDH mutations)	NOXA (PMAIP1), ATF4, BCL-2, MCL-1	NCT05401097	Phase 1/2
Azacitidine combined with venetoclax and bleximenib	Relapsed/refractory (R/R) or newly diagnosed, intensive chemotherapy-ineligible acute myeloid leukemia (AML)	NPM1 mutations (NPM1m) KMT2A rearrangements (KMT2Ar),	NCT06852222	Phase 3

#### 4.2.1 All-trans retinoic acid

Acute Promyelocytic Leukemia (APL) is primarily driven by a chromosomal translocation, t(15;17), which fuses the promyelocytic leukemia (PML) gene with the retinoic acid receptor alpha (RARα) gene. This fusion impairs critical cellular processes, including senescence, RNA processing, and apoptosis (99, 100, 145). The PML-RARα fusion protein, which preserves essential RARα components, serves as the primary oncogenic driver in APL (99) (Table 2).

**Table 2:** Key biomarkers in acute myeloid leukemia (AML) with their types, clinical significance, and associated subtypes or features.

Biomarker	Type	Clinical significance	Associated AML subtypes/features
PML-RARA	Fusion gene (t(15;17))	Diagnostic marker for APL; excellent response to ATRA + ATO	APL (M3 subtype)
AML1-ETO	Fusion gene (t(8;21))	Good prognosis; altered if c-Kit mutated	AML-M2, core binding factor AML
CD34	Surface glycoprotein marker	Linked to unfavourable prognosis, less complete response, and increased relapse risk.	M0, M1, M2; mostly absent in APL
*NPM1*	Nucleolar protein mutation	Favorable if *FLT3*-ITD−; useful for MRD monitoring	M4/M5, Normal Karyotype AML
*FLT3*-ITD/TKD	Tyrosine kinase mutations	Poor prognosis with *FLT3*-ITD; targetable with TKIs	Normal karyotype AML; co-mutates with *NPM1*
TP53	Tumor suppressor mutation	Very poor prognosis; high relapse; drug resistance	Complex karyotype AML, therapy-related AML
IDH1/2	Metabolic enzyme mutations	Epigenetic dysregulation; emerging MRD markers; drug-targetable	Normal karyotype AML; often co-mutated with *NPM1*

This table summarizes diagnostic, prognostic, and therapeutic markers commonly used in AML risk stratification and treatment guidance.

The therapeutic effects of all-trans retinoic acid (ATRA) in APL by targeting nuclear receptors, specifically retinoic acid receptor alpha (RARα) and retinoid X receptor (RXR), which function as transcription factors activated by ligands. These receptors modulate gene expression by binding to retinoic acid response elements (RAREs) on DNA (101). Without retinoic acid, RARα/RXR heterodimers attract corepressor complexes, such as SMRT and NcoR1, which associate with histone deacetylases (HDACs), keeping chromatin in a suppressed state and inhibiting genes critical for cell differentiation. In APL, the PML-RARα fusion protein disrupts normal retinoid signaling by strongly recruiting these corepressor complexes, leading to sustained gene silencing and impaired myeloid differentiation. To overcome this, high ATRA concentrations (around 10^−6^ M) are required to induce a structural change in the receptor complex, releasing corepressors and engaging coactivators to restore expression of genes essential for granulocytic development (102, 103). Additionally, ATRA facilitates the breakdown of the PML-RARα fusion protein through proteasome- and autophagy-dependent mechanisms, eliminating the primary oncogenic driver of APL (104). Thus, ATRA’s dual mechanism in APL involves reactivating differentiation-related gene expression and promoting PML-RAR*α* degradation, both of which are vital for restoring normal blood cell production (105).

#### 4.2.2 Arsenic trioxide: dual role in differentiation and apoptosis

Arsenic trioxide (ATO) engages with both PML and the PML-RARα fusion protein, initiating their post-translational modification by sumoylation and ubiquitination, thereby designating them for proteasomal destruction. Through this procedure, PML nuclear bodies’ structure and functionality are restored activates p53/p73-dependent signaling pathways, and promotes leukemic cell apoptosis and partial myeloid differentiation (106).

Although, initially introduced as a salvage option for relapsed APL, Arsenic trioxide has since shown strong therapeutic efficacy in clinical trials, establishing its role as a first-line agent, particularly in patients with low- and intermediate-risk disease profiles (107).

ATO Restores PML Bodies and Induces Apoptosis: Arsenic trioxide (ATO) binds directly to the PML and PML-RARα fusion proteins, triggering their post-translational modification via sumoylation and polyubiquitination. These modifications mark the fusion protein for proteasomal degradation. As a result, normal PML nuclear bodies (NBs) are reassembled, and tumor suppressor pathways involving p53 and p73 are reactivated. The downstream effect is either differentiation or apoptosis of APL cells, depending on the drug concentration (106, 108).

At low doses (0.1–0.5 μmol/L), arsenic trioxide (ATO) causes leukemic blasts to differentiate into granulocytes, whereas at higher concentrations (0.5–2 μmol/L), it triggers the intrinsic mitochondrial pathway of apoptosis (98). Beyond these effects, ATO supports the elimination of leukemic cells by enhancing differentiation, apoptosis, autophagy, and extracellular trap formation (ETosis). These mechanisms contribute to the destruction of leukemia-initiating cells (LICs), which are often resistant to conventional therapies and linked to disease relapse. ATO-driven autophagy promotes the breakdown of oncogenic proteins and damaged organelles, while ETosis a type of cell death that is preprogrammed involving the release of chromatin structures further supports the clearance of leukemic blasts and stem-like cells through immunogenic pathways (109–111, 149).

#### 4.2.3 Enasidenib: reversing epigenetic blockade in *IDH2-*mutant AML

Enasidenib, an oral targeted therapy, selectively inhibits the dysfunctional mutant *IDH2* enzyme, decreasing the production of the oncometabolite 2-hydroxyglutarate (2-HG) and promoting differentiation of leukemic precursor cells. It gained FDA approval in August 2017 for treating adults with relapsed or refractory AML harbouring *IDH2* mutations (112).

##### 4.2.3.1 *IDH2* inhibition and epigenetic reversal

Mutations in the IDH2 gene result in a defective enzyme with altered enzymatic activity, converting α-ketoglutarate (α-KG) into the atypical metabolite 2-HG. Excessive 2-HG disrupts α-KG-dependent enzymes that regulate cellular epigenetic states, leading to hypermethylation of histones and DNA. This epigenetic alteration silences genes essential for normal blood cell development, hindering hematopoietic differentiation (113, 114). Enasidenib specifically targets this mutant IDH2 enzyme, significantly reducing 2-HG levels in preclinical models and clinical trials, thereby alleviating the epigenetic block and enabling myeloblasts to mature into functional blood cells (113). Clinical studies demonstrated that Enasidenib treatment resulted in a median maximum 2-HG reduction of 97%, compared to only 12% with conventional care regimens (CCR), confirming its direct action on the molecular target. Additionally, patients achieving complete remission (CR) exhibited a substantial decrease in the IDH2 variant allele frequency (VAF) a metric of mutation-carrying leukemic cells with a median reduction of 74%, while those with partial or no response showed minimal reductions, indicating that stronger molecular responses correlate with improved clinical outcomes (115).

#### 4.2.4 Ivosidenib (AG-120): targeting mutant *IDH1* to promote myeloid maturation

##### 4.2.4.1 Targeting mutant metabolism

In cancer cells, mutations at the IDH1 enzyme’s arginine-132 (R132) position confer a unique, harmful activity that results in the formation of the aberrant metabolite D-2-hydroxyglutarate (2-HG). Increased 2-HG levels interfere with proper cell development by disrupting epigenetic control. The study introduces AG-120 (Ivosidenib), a selective inhibitor of mutant *IDH1* that significantly lowers 2-HG levels in tumor models and encourages the differentiation of cells generated from AML patients in ex vivo tests (116).

##### 4.2.4.2 *IDH1* blockade promotes myeloid maturation

AG-120 demonstrated strong activity in reducing tumor levels of the oncometabolite 2-HG in a mouse model (HT1080 xenografts). A solitary oral administration of AG-120 at dosages of 50 or 150 mg/kg resulted in fast decreases in tumour 2-HG levels, with maximum reductions of 92 and 95.2% recorded at 12 h post-dosing. The levels progressively reverted to baseline within 48–72 h, signifying that the suppression by AG-120 is reversible (113, 117–119). IDH1 mutations are known to impair normal cell differentiation through epigenetic and metabolic alterations. Consistent with this, AG-120 was found to promote differentiation in AML patient-derived cells carrying *IDH1* mutations (R132H or R132C) when tested ex vivo. Treated cells showed enhanced colony formation, increased expression of differentiation surface markers, and a greater proportion of mature myeloid cells. No such effect was observed in cells with wild-type *IDH1* (120).

#### 4.2.5 Azacitidine: epigenetic modulator with noncanonical apoptosis-inducing effects

Azacitidine (5-Aza) triggers programmed cell death in AML cells via a recently discovered mechanism distinct from its traditional epigenetic role, specifically by upregulating the transcription of NOXA, a pro-apoptotic BH3-only protein that promotes apoptosis (121). Upon entering cells, 5-Aza incorporates into both DNA and RNA. Its integration into DNA depletes DNA methyltransferase (DNMT) enzymes, resulting in global DNA hypomethylation and the reactivation of previously silenced genes (122, 123). However, 80 to 90% of the intracellular 5-Aza is integrated into RNA, this RNA-directed incorporation is believed to contribute to non-epigenetic effects (124) (Glover and Leyland-Jones, 1987) including rapid activation of apoptosis pathways (125, 126). Importantly, clinical responses to 5-Aza in myeloid malignancies are not consistently predicted by the extent of DNA demethylation. This supports the idea that non-DNA-related mechanisms, such as RNA-mediated or stress response–linked pathways, play a critical therapeutic role (127).

##### 4.2.5.1 NOXA activation and stress-induced apoptosis

Earlier research proposed that 5-Azacitidine does not trigger BH3-only proteins by integrating into DNA but instead by inserting into RNA and blocking protein synthesis (128–130). which may activate the integrated stress response (ISR) (124). The integrated stress response (ISR) pathway is activated under severe cellular stress, with its primary regulator, ATF4, driving apoptosis by upregulating pro-apoptotic genes (131).

ATF4 stimulates PMAIP1 expression by binding to its promoter, and its levels increase significantly in AML cells following 5-Aza (Azacitidine) treatment (132, 133). Post-treatment with 5-Aza, NOXA’s binding affinity for BCL-2 and MCL-1 proteins rises in a dose-dependent manner, notably in Kasumi-1 and MV4-11 AML cell lines. Deletion of the PMAIP1 gene reduces the synergistic effects of venetoclax and 5-Aza, highlighting NOXA’s critical role in venetoclax efficacy, whether used alone or in combination therapy (121).

#### 4.2.6 Menin inhibitors: restoring differentiation in KMT2A-rearranged and *NPM1*-mutated AML

A self-renewal program is enforced and myeloid differentiation is inhibited by Menin, a scaffold protein encoded by MEN1, which interacts with KMT2A (MLL) fusion proteins to sustain the transcription of HOXA cluster genes and MEIS1. Particularly in AML with KMT2A rearrangement and *NPM1* mutation, inhibiting the menin–KMT2A interaction results in a downregulation of HOX/MEIS1 expression and a restoration of hematopoietic maturation (134–136, 147).

Preclinical studies have demonstrated that small-molecule menin inhibitors, like revumenib (SNDX-5613) and ziftomenib (KO-539), effectively reduce leukemic proliferation and increase differentiation markers (CD11b, CD14) in xenograft models (134, 136, 137). Revumenib was tested in the AUGMENT-101 trial (NCT04065399) for relapsed/refractory AML. The results showed an overall response rate (ORR) of approximately 65% and a complete remission/complete remission with partial hematologic recovery (CR/CRh) rate of approximately 23%, with responses being more enriched in patients who had *NPM1* mutations or KMT2A rearrangements (138).

Some patients were able to move forward with hematopoietic stem cell transplantation with CR/CRh of 23.4% and ORR of 46.9% in a dedicated *NPM1*-mutant group of the same experiment (11). Similarly, ziftomenib’s KOMET-001 study (NCT04067336) showed clinically significant effectiveness in *NPM1*-mutant AML, with ORR of approximately 45% and CR/CRh rates of approximately 35%, suggesting the possibility of menin inhibition as a differentiation-inducing tactic (139). New findings also point to secondary *MEN1* mutations as a source of resistance, highlighting the necessity of sensible combinations with chemotherapy, *IDH* inhibitors, or venetoclax (Steger et al., 2023).

#### 4.2.7 Combination strategies

Venetoclax combined with hypomethylating drugs (e.g., azacitidine) attains elevated complete remission (CR) and complete remission with incomplete blood count recovery (CRi) rates (66–91%) in newly diagnosed NPM1-mutated acute myeloid leukemia (AML), frequently resulting in profound molecular responses, surpassing conventional therapy according to real-world data. Novel combinations encompass venetoclax combined with quizartinib (CRc 52.5% in unfit FLT3-mutated patients), menin inhibitors paired with venetoclax or FLT3 inhibitors to achieve synergy in NPM1/FLT3 dual-mutated cases, and ongoing trials such as azacitidine combined with venetoclax and bleximenib (NCT06852222) (140, 141). Although, these medicines target unfavorable outcomes in high-risk profiles such as FLT3-ITD with elevated allelic ratio alongside NPM1 or TP53/IDH1/2 co-mutations; for instance, quizartinib combined with chemotherapy produces more profound remissions and reduces relapse risk in frontline FLT3-mutated acute myeloid leukemia (AML). Integrating them boosts translational significance by boosting relapse/refractory survival and minimal residual disease clearance (142).

#### 4.2.8 Limitations and future direction

Clinical trials with triplet regimens such as azacitidine, venetoclax, and bleximenib demonstrate elevated initial response rates (e.g., 82% overall response rate, 59% complete clinical response in relapsed/refractory NPM1-mutated acute myeloid leukemia); nevertheless, durability is a significant restriction, with median response lengths sometimes below 12 months and elevated relapse rates following remission. Responses exhibit rapid onset but limited durability; frontline unsuited individuals attained 75% complete clinical response however encounter early relapses due to clonal evolution, in contrast to the deeper minimal residual disease negativity observed in certain venetoclax doublets, underscoring the necessity for maintenance treatments (141). Second-generation FLT3 inhibitors and menin inhibitors target critical mutations, whilst venetoclax-based triplet therapies, such as azacitidine + venetoclax + bleximenib, demonstrate potential in NPM1-mutated AML, albeit with durability concerns (143). Future research should focus on phase 3 outcomes from studies such as NCT06852222 to validate triplet superiority, investigate quadruplets incorporating FLT3 inhibitors for dual-mutated AML, and establish MRD-guided maintenance with menin inhibitors beyond remission. Surmounting resistance necessitates biomarker-guided approaches aimed at HOX reactivation or BCL-2 evasion, in conjunction with innovative medicines such as next-generation epigenetic modulators. Long-term objectives encompass frontline integration for unsuitable patients and the optimization of HSCT to prolong event-free survival beyond existing thresholds.

## 5 Conclusion

The molecular complexity, clonal heterogeneity, and treatment resistance of acute myeloid leukemia (AML) make it an extremely difficult disease to treat. The 7 + 3 induction regimen has long been the mainstay of treatment, however it has limited therapeutic potential, especially for elderly patients and those with genetically predisposed to unfavourable outcomes. However, the use of targeted medicines and, more significantly, the return of differentiation-based techniques have revolutionized AML therapy throughout the past 20 years.

It was demonstrated that reversing differentiation arrest might turn a leukemia that is extremely deadly into one of the most treatable subtypes by the historic success of ATRA and ATO in treating acute promyelocytic leukemia (APL). Using this paradigm as a foundation, more recent treatments such IDH1/2 inhibitors have shown that they can cause differentiation in genetically specified subsets of AML. By disrupting the epigenetic mechanisms that promote leukemic stemness, menin inhibitors, which are presently undergoing advanced clinical trials, have demonstrated encouraging efficacy in treating AML with *NPM1* mutations and KMT2A rearrangements (148). Azacitidine and other epigenetic modulators highlight the interaction between transcriptional regulation, metabolism, and differentiation.

However, there are still major obstacles. Leukemic stem cell survival, subsequent resistance mutations, and relapse continue to be significant obstacles. Personalized therapy is hampered by the absence of reliable prognostic biomarkers. Reasonable medication combinations, the incorporation of differentiation agents with immune-based and targeted therapies, and precision genomic profiling to direct customized treatment will be essential for future advancements. To sum up, differentiation therapy has developed from a specialized success in APL to a key and growing area of study in AML. It provides an effective, less harmful, and supplementary approach to chemotherapy and targeted therapy by specifically addressing the underlying pathologic process of maturation arrest. Differentiation therapy has the potential to revolutionize AML treatment and greatly enhance patient outcomes with further innovation and biomarker-driven strategies.
